# Supplementary material for: PepQuery2 democratizes public MS proteomics data for rapid peptide searching
Source: Nat Commun. 2023 Apr 18;14:2213. doi: 10.1038/s41467-023-37462-4 (PMC10113256; doi:10.1038/s41467-023-37462-4)
Supplement: Supplementary file 1 — Supplementary Information [file 41467_2023_37462_MOESM1_ESM.pdf]

Supplementary information

PepQuery2 democratizes public proteomics data for rapid peptide matching

Bo Wen[1,2,3], *Bing Zhang*[1,2#]

[1]Lester and Sue Smith Breast Center, Baylor College of Medicine, Houston, TX 77030, USA

[2]Department of Molecular and Human Genetics, Baylor College of Medicine, Houston, TX 77030, USA

[3]Present address: Department of Genome Sciences, University of Washington, Seattle, WA 98195, USA

# Correspondence should be addressed to B.Z. (bing.zhang@bcm.edu).

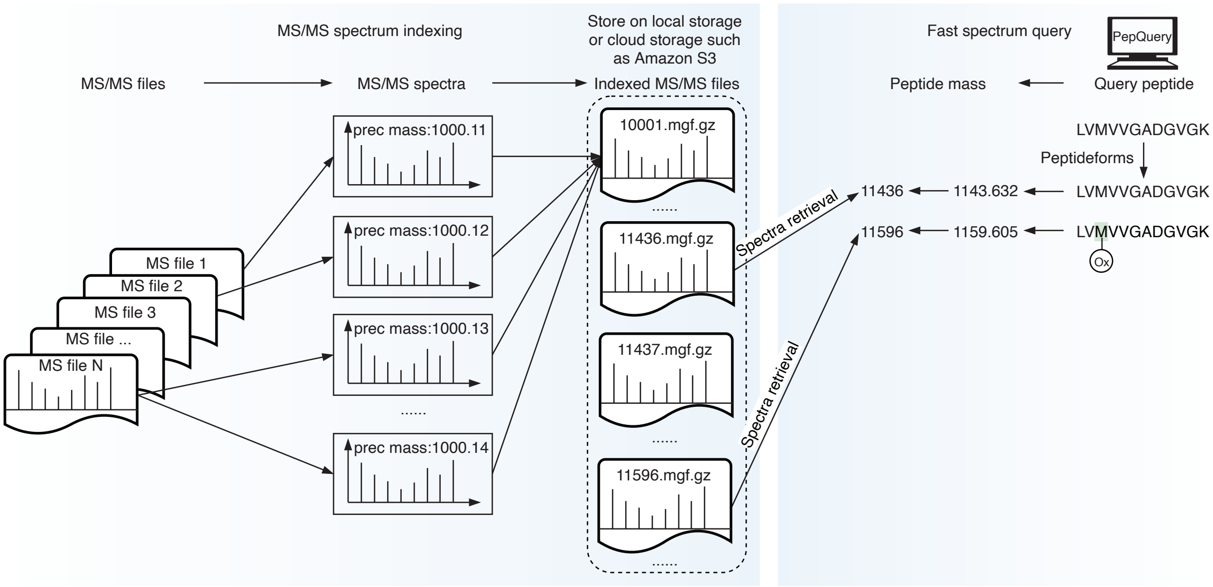

Supplementary Figure 1: MS/MS data indexing and fast spectrum query. The indexed MS/MS files for PepQueryDB are stored on cloud storage and can be accessed in any computer with internet connection.

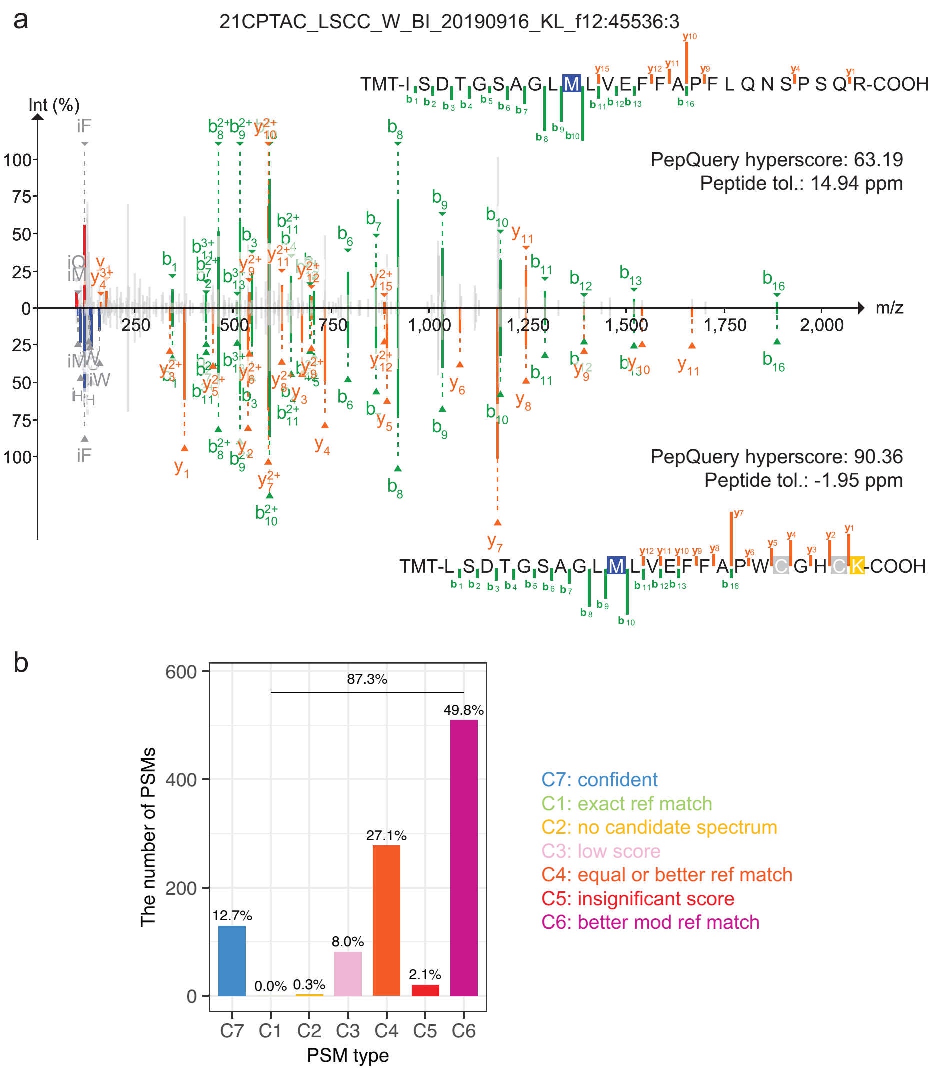

Supplementary Figure 2: W2F peptide validation. (a) The spectrum originally matched to a W2F peptide has better match to a reference peptide containing amino acid W in PepQuery2 validation. (b) PepQuery2 classified PSMs identified by a reanalysis supporting novel peptides resulted from W to F substitution into seven categories as described in Figure 1, and only C7 PSMs passed the validation.

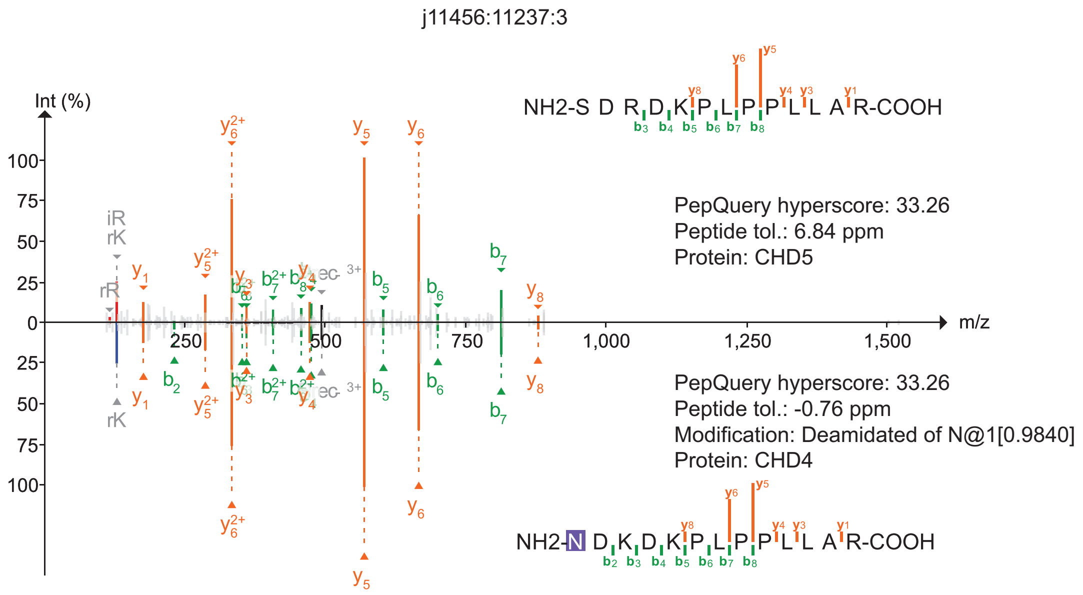

Supplementary Figure 3: Validation result for prey protein CHD5 previously reported in an AP-MS experiment using HDAC1 as the bait. An originally reported spectrum identifying CHD5 was found by PepQuery2 to have equally good match to a peptide from CHD4 with deamidation, but the peptide mass tolerance was much smaller for the CHD4 peptide.

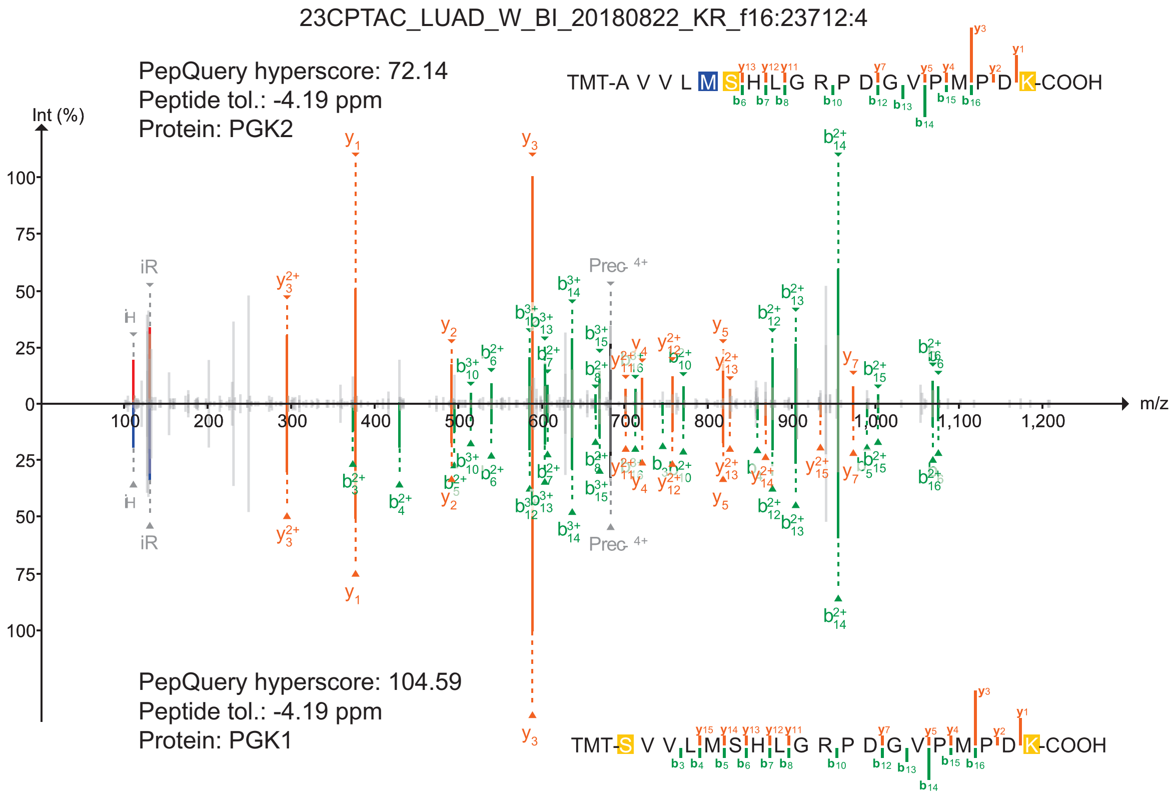

Supplementary Figure 4: An example PSM from the LUAD dataset for a PGK2 peptide. The spectrum was identified to have better match to a peptide from PGK1.

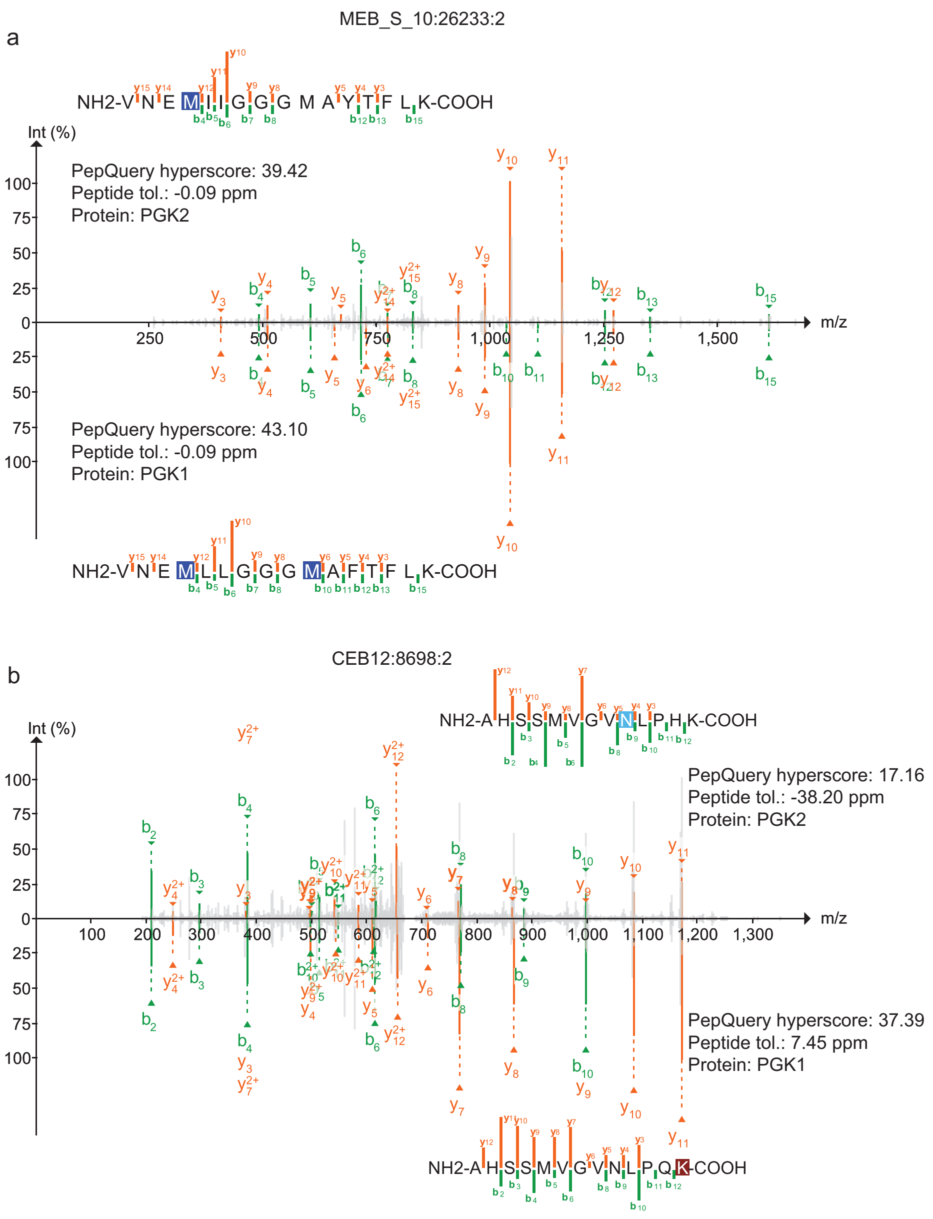

Supplementary Figure 5: Two representative PSMs from the colorectal cancer dataset MSV000088431 for PGK2 peptides. The spectra were found to have better matches to peptides from PGK1.

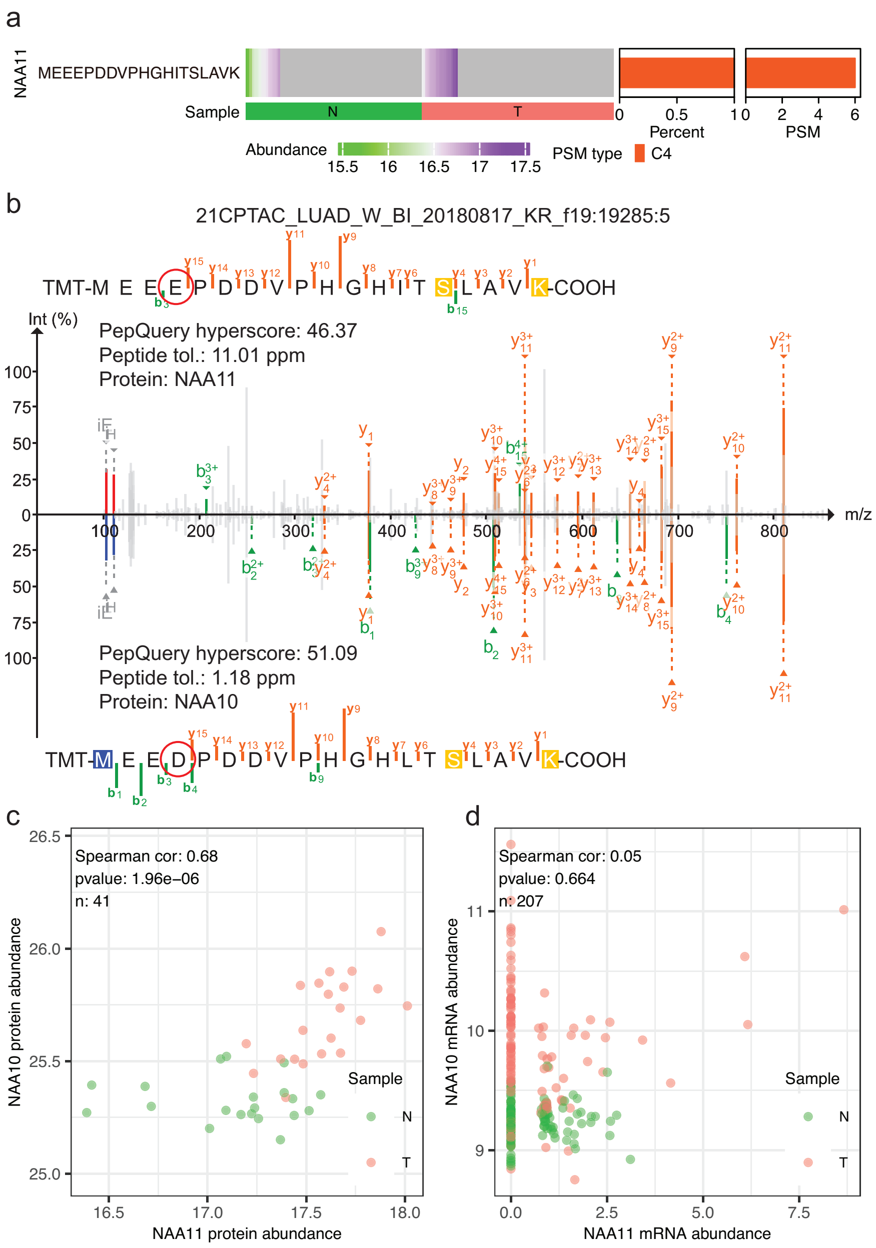

Supplementary Figure 6: NAA11 peptide validation using PepQuery2. (a) All PSMs previously reported to identifying NAA11 failed PepQuery2 validation (classified as C4). (b) A representative PSM from the LUAD dataset for the NAA11 peptide. The spectrum was identified to have a better match to a peptide from NAA10. (c) Protein abundance correlation between NAA11 and NAA10 in the LUAD dataset. (d) mRNA abundance correlation between NAA11 and NAA10 in the LUAD dataset. For c and d, only samples with non-missing and non-zero values in both samples were considered when calculating the spearman correlation.
