# Supplementary material for: PepQuery2 democratizes public MS proteomics data for rapid peptide searching
Source: Nat Commun. 2023 Apr 18;14:2213. doi: 10.1038/s41467-023-37462-4 (PMC10113256; doi:10.1038/s41467-023-37462-4)
Supplement: Supplementary file 3 — Description of Additional Supplementary Files [file 41467_2023_37462_MOESM3_ESM.pdf]

Description of Additional Supplementary Files

Title: Supplementary Data 1.
Description: A list of MS/MS datasets included in the PepQueryDB.

Title: Supplementary Data 2.
Description: Identified PSMs for the KRAS G12D mutant peptide.

Title: Supplementary Data 3.
Description: Results for validating novel peptides with W to F substitution.

Title: Supplementary Data 4.
Description: The list of nuORF-derived peptides identified in healthy tissues and associated information.

Title: Supplementary Data 5.
Description: The list of identified missing proteins and associated information.
