# Supplementary material for: PepQuery2 democratizes public MS proteomics data for rapid peptide searching
Source: Nat Commun. 2023 Apr 18;14:2213. doi: 10.1038/s41467-023-37462-4 (PMC10113256; doi:10.1038/s41467-023-37462-4)
Supplement: Supplementary file 9 — Reporting Summary [file 41467_2023_37462_MOESM9_ESM.pdf]

nature portfolio

Corresponding author(s): Bing Zhang

Last updated by author(s): Feb 26, 2023

Reporting Summary

Nature Portfolio wishes to improve the reproducibility of the work that we publish. This form provides structure for consistency and transparency in reporting. For further information on Nature Portfolio policies, see our Editorial Policies and the Editorial Policy Checklist.

Statistics

For all statistical analyses, confirm that the following items are present in the figure legend, table legend, main text, or Methods section.

n/a	Confirmed	
☐	☒	The exact sample size (*n*) for each experimental group/condition, given as a discrete number and unit of measurement
☒	☐	A statement on whether measurements were taken from distinct samples or whether the same sample was measured repeatedly
☐	☒	The statistical test(s) used AND whether they are one- or two-sided<br>*Only common tests should be described solely by name; describe more complex techniques in the Methods section.*
☒	☐	A description of all covariates tested
☒	☐	A description of any assumptions or corrections, such as tests of normality and adjustment for multiple comparisons
☐	☒	A full description of the statistical parameters including central tendency (e.g. means) or other basic estimates (e.g. regression coefficient) AND variation (e.g. standard deviation) or associated estimates of uncertainty (e.g. confidence intervals)
☐	☒	For null hypothesis testing, the test statistic (e.g. *F*, *t*, *r*) with confidence intervals, effect sizes, degrees of freedom and *P* value noted<br>*Give P values as exact values whenever suitable.*
☒	☐	For Bayesian analysis, information on the choice of priors and Markov chain Monte Carlo settings
☒	☐	For hierarchical and complex designs, identification of the appropriate level for tests and full reporting of outcomes
☒	☐	Estimates of effect sizes (e.g. Cohen's *d*, Pearson's *r*), indicating how they were calculated

Our web collection on statistics for biologists contains articles on many of the points above.

Software and code

Policy information about availability of computer code

Data collection	No software was used for data collection
Data analysis	Both the command line version and the web version of PepQuery2 are available at http://www.pepquery.org. The source code of PepQuery2 is available at https://github.com/bzhanglab/PepQuery. We used AutoRT for retention time prediction. We used FragPipe (v18.0) for reanalysis of the LSCC data. FragPipe (v18.0) was powered by the MSFragger15 (v3.4) search engine and the Philosopher toolkit39 (v4.4).

For manuscripts utilizing custom algorithms or software that are central to the research but not yet described in published literature, software must be made available to editors and reviewers. We strongly encourage code deposition in a community repository (e.g. GitHub). See the Nature Portfolio guidelines for submitting code & software for further information.

Data

Policy information about availability of data

All manuscripts must include a data availability statement. This statement should provide the following information, where applicable:

- Accession codes, unique identifiers, or web links for publicly available datasets
- A description of any restrictions on data availability
- For clinical datasets or third party data, please ensure that the statement adheres to our policy

The MS/MS datasets indexed in PepQueryDB were downloaded from PDC (https://pdc.cancer.gov/pdc/), PRIDE (https://www.ebi.ac.uk/pride/), or MassIVE (https://massive.ucsd.edu). Detailed dataset information is available in Supplementary Data 1. The KRAS mutations detected at the DNA level for CPTAC tumor samples were

downloaded from LinkedOmics (http://linkedomics.org). The human reference proteins used in the reanalysis of the CPTAC LSCC global proteome dataset were downloaded from UniProt on 12/19/2022. The AP-MS data from BioPlex 3.024 was downloaded from MassIVE through the accession number MSV000088555. The human reference proteins used in the analysis of the AP-MS data were downloaded from UniProt on 04/26/2022. The MS/MS data from the proteome of metastatic cells in colorectal cancer27 was accessed from MassIVE through the accession number MSV000088431, and the protein database from GENCODE Human release 34 was used as the reference database for this analysis. The protein sequences of the nuORFs were downloaded from MassIVE (https://massive.ucsd.edu) through the accession number MSV000084787. The raw MS/MS data from the analyzing 29 healthy human tissues were downloaded from PRIDE through the accession number PXD010154, and the protein database from GENCODE Human release 34 was used as the reference database for this analysis. The missing proteins were downloaded from neXtProt (https://www.nextprot.org/, 04/14/2022). For the XBP1 analysis, the SwissProt human protein database including protein isoforms (05/17/2022) was used as the reference database.

Human research participants

Policy information about studies involving human research participants and Sex and Gender in Research.

Reporting on sex and gender	N/A
Population characteristics	N/A
Recruitment	N/A
Ethics oversight	N/A

Note that full information on the approval of the study protocol must also be provided in the manuscript.

Field-specific reporting

Please select the one below that is the best fit for your research. If you are not sure, read the appropriate sections before making your selection.

☒ Life sciences ☐ Behavioural & social sciences ☐ Ecological, evolutionary & environmental sciences

For a reference copy of the document with all sections, see nature.com/documents/nr-reporting-summary-flat.pdf

Life sciences study design

All studies must disclose on these points even when the disclosure is negative.

Sample size	No new data generation. Sample sizes were from the original publications, and they were sufficient for all statistical testes performed.
Data exclusions	none
Replication	No new data generation, this study reanalyzes previously published data.
Randomization	Randomization is not applicable because there was no new experiments.
Blinding	Blinding is not applicable because there was no new experiments.

Reporting for specific materials, systems and methods

We require information from authors about some types of materials, experimental systems and methods used in many studies. Here, indicate whether each material, system or method listed is relevant to your study. If you are not sure if a list item applies to your research, read the appropriate section before selecting a response.

Materials & experimental systems

n/a	Involved in the study
☒	☐ Antibodies
☒	☐ Eukaryotic cell lines
☒	☐ Palaeontology and archaeology
☒	☐ Animals and other organisms
☒	☐ Clinical data
☒	☐ Dual use research of concern

Methods

n/a	Involved in the study
☒	☐ ChIP-seq
☒	☐ Flow cytometry
☒	☐ MRI-based neuroimaging
